# Identification of Ecdysone Hormone Receptor Agonists as a Therapeutic Approach for Treating Filarial Infections

**DOI:** 10.1371/journal.pntd.0004772

**Published:** 2016-06-14

**Authors:** Amruta S. Mhashilkar, Sai L. Vankayala, Canhui Liu, Fiona Kearns, Priyanka Mehrotra, George Tzertzinis, Subba R. Palli, H. Lee Woodcock, Thomas R. Unnasch

**Affiliations:** 1 Department of Global Health, College of Public Health, University of South Florida, Tampa, Florida, United States of America; 2 Department of Chemistry, University of South Florida, Tampa, Florida, United States of America; 3 New England Biolabs, Ipswich, Massachusetts, United States of America; 4 Department of Entomology, University of Kentucky, Lexington, Kentucky, United States of America; New York Blood Center, UNITED STATES

## Abstract

**Background:**

A homologue of the ecdysone receptor has previously been identified in human filarial parasites. As the ecdysone receptor is not found in vertebrates, it and the regulatory pathways it controls represent attractive potential chemotherapeutic targets.

**Methodology/ Principal Findings:**

Administration of 20-hydroxyecdysone to gerbils infected with *B*. *malayi* infective larvae disrupted their development to adult stage parasites. A stable mammalian cell line was created incorporating the *B*. *malayi* ecdysone receptor ligand-binding domain, its heterodimer partner and a secreted luciferase reporter in HEK293 cells. This was employed to screen a series of ecdysone agonist, identifying seven agonists active at sub-micromolar concentrations. A *B*. *malayi* ecdysone receptor ligand-binding domain was developed and used to study the ligand-receptor interactions of these agonists. An excellent correlation between the virtual screening results and the screening assay was observed. Based on both of these approaches, steroidal ecdysone agonists and the diacylhydrazine family of compounds were identified as a fruitful source of potential receptor agonists. In further confirmation of the modeling and screening results, Ponasterone A and Muristerone A, two compounds predicted to be strong ecdysone agonists stimulated expulsion of microfilaria and immature stages from adult parasites.

**Conclusions:**

The studies validate the potential of the *B*. *malayi* ecdysone receptor as a drug target and provide a means to rapidly evaluate compounds for development of a new class of drugs against the human filarial parasites.

## Author Summary

The human filarial parasites are the causative agents of two neglected tropical diseases targeted for elimination by the international community. The current elimination programs rely upon the mass distribution of a limited number of drugs, leaving the programs open to failure in the event that resistance develops. Thus, there is a critical need for novel chemotherapeutic agents to supplement the current arsenal. The filarial parasites are ecdysozoans, whose developmental processes are controlled by a master regulator, the ecdysone receptor. Here we validate the potential of the filarial ecdysone receptor as a chemotherapeutic target and report the development of high throughput and virtual screening assays that may be used to compounds that target it.

## Introduction

Diseases caused by the human filarial parasitic nematodes are a significant public health problem faced by developing countries. Recent reports estimate that there are over 140 million individuals suffering from human filarial parasites in over 80 countries worldwide. Approximately 1 billion people are at risk for contracting the filarial infections [1]. Lymphatic filariasis (caused by infection with *Brugia malayi*, *Brugia timori* or *Wuchereria bancrofti*) and onchocerciasis (caused by infection with *Onchocerca volvulus*) are severely debilitating diseases, together resulting in a loss of 5.7 million disability adjusted life years [2].

Both lymphatic filariasis (LF) and onchocerciasis have been designated as neglected tropical diseases by the international community, and both have been targeted for elimination in the London Declaration on Neglected Tropical Diseases (NTDs) [3].

The efforts to eliminate LF and other NTDs have generally relied on mass drug administration (MDA) programs. Currently, a combination of ivermectin plus albendazole (ALB) or diethylcarbamazine (DEC) plus ALB are employed for MDA for filariasis (depending upon the region), while ivermectin (IVM) monotherapy is the standard for MDA targeting onchocerciasis. However, recent clinical trials with a single-dose triple-drug therapy of DEC, IVM and ALB [4], and twice-yearly treatment with ALB + IVM [5] have shown very promising results when compared to the two-drug regimens currently used by traditional filariasis MDA programs.

Though MDA programs have on the whole been well formulated and executed, they face potential challenges that may threaten their ultimate success. These include the development of resistance to the limited number of drugs that are deployed by the MDA programs [6] and limits on the use of MDA in certain areas. For example, DEC + ALB is used to treat LF by MDA programs throughout India, South America and Southeast Asia [7,8]. However it cannot be used in Africa, as it causes severe ocular side effects and systemic complications in individuals co-infected with *O*. *volvulus*, the causative agent of onchocerciasis, a disease that is endemic throughout most of sub-Saharan Africa [9,10]. Similarly, ivermectin, which is the only drug used by the MDA programs worldwide to treat onchocerciasis, cannot be easily used in much of Central Africa. This area is endemic for the eyeworm *Loa loa*, and treatment of *L*. *loa* infected individuals with ivermectin can result in severe neurological reactions, including coma and death [11]. Thus, new treatments are desperately needed for these infections.

Insect growth regulators (IGRs) have been used in veterinary medicine to treat ectoparasites like ticks, fleas, lice and mites. The IGRs interfere with the larval molt and embryogenesis by targeting one of two pathways: (i) chitin inhibitors acting on cuticle synthesis and degradation, and (ii) hormonal (ecdysone and juvenile hormone) analogs [12]. Ecdysteroids are master regulators of development in arthropods, and are thought to also play a central role in controlling development in other organisms in which molting is a central feature of the life cycle (the ecdysoans) [13]. In insects, molting and other developmental processes (including embryogenesis) are controlled through variation in the levels of the molting hormones, or ecdysteroids, which induce molting, and the juvenile hormones, which inhibit molting [14,15]. This process is mediated through a heterodimer of the ecdysone receptor (EcR) and ultraspiracle, the homologue of retinoid X receptor (RXR) which controls the transcriptional activity of the developmental genes regulating molting and metamorphosis [16]. The fact that ecdysis is a central developmental pathway in insects and is absent in vertebrates has made it an attractive target for the development of compounds that might act selectively against invertebrates [17,18]. Thus, the agricultural industry has targeted the EcR in pesticide development, as insects represent one of the largest classes of ecdyzoans on Earth. This high degree of species-specific activity makes the EcR an excellent target for pest management. For example, tebufenozide has insecticidal activity against lepidopteran pests but shows low activity against the hymenopteran insects [18].

Several lines of evidence suggest that like insects, many developmental processes in parasitic nematodes may be controlled in part by ecdysteroids. First, ecdysone and related compounds have been found in many parasitic nematodes, including *O*. *volvulus* (for a review, see [19]). Second, ecdysteroid levels vary during nematode development. In *Ascaris suum*, the level of 20-hydroxyecdysone (20E) has been shown to peak just before the third and fourth larval molts, similar to the temporal pattern seen during the larval molts in insects [20]. Third, parasitic nematodes are capable of catabolizing ecdysone [21]. Fourth, ecdysteroids have been shown to have developmental effects on parasitic nematodes. For example, ecdysone was shown to stimulate microfilarial release in *Brugia pahangi* and to promote embryogenesis in ovaries of *D*. *immitis* adult females [22]. Fifth, a homolog of the EcR has been identified and proven to be functional in *Brugia malayi* [13,23]. Homologues of the EcR have also been identified in the genomes of other human filarial parasites. Previous studies have demonstrated that treatment of the filarial worm with ecdysteroids can affect embryogenesis [23], molting [24] and viability of adult parasites [25], suggesting that the EcR might represent an attractive chemotherapeutic target against filarial infections. In this study, we validate the *Brugia malayi* ecdysone receptor (*Bma*EcR) as a chemotherapeutic target and report progress towards the development of a high-throughput screening assay to identify agonists and antagonists against the *Bma*EcR. We also report the development of a homology model of the *Bma*EcR that may be useful in optimizing leads identified by high throughput and *in-silico* approaches.

## Materials and Methods

### Ethics statement

All animal work was conducted according to guidelines outlined by the National Institutes of Health Office of Laboratory Animal Welfare, and was approved by The Institutional Animal Care and Use Committee (IACUC) of the University of South Florida, under protocol IS00000261.

### Infection and 20-hydroxyecdysone (20E) treatment of gerbils

*Brugia malayi* infective larvae (L3) were obtained from the Filariasis Research Reagent Resource Center (FR3) at the University of Georgia, USA. A total of four male gerbils were infected intraperitoneally with 150 L3s each, under aseptic conditions. The experimental animals (n = 2) received 20E (Adipogen, CA) at a dose of 5mg/kg/day/animal for 150 days. The control animals (n = 2) received ethanol, the vehicle used for dissolving 20E. To avoid the potential for trauma induced by having to carry out multiple gavages on each animal, Alzet mini-osmotic pumps (Model number 2006) were used to provide a steady supply of 20E in ethanol or ethanol alone to the infected animals. The pumps were surgically implanted on the dorsal aspect of the gerbils two days post-infection. The pumps were replaced every 42 days following the manufacturer’s instructions. Animals were euthanized after 150 days post-infection and peritoneal gavage was performed to recover microfilariae. Necropsy was then conducted to recover adult worms from the peritoneal cavity.

### Constructs for creating stable cell line

Previous studies utilized a system in which plasmids were transiently transfected into NIH3T3 cells (American Type Culture Collection- www.atcc.org; CRL-1658) [13]. However, in our studies the plasmids were transfected into HEK293 cells (American Type Culture Collection- www.atcc.org; CRL-1573) as we observed improved growth and enhanced assay characteristics when these cells were used. To adapt the transient transfection assay into a high throughput screen, a stable mammalian cell line was developed, in which all three of the necessary constructs were integrated into the HEK293 genome, employing the Gateway recombination system (Invitrogen, CA). In brief, three cassettes were constructed. The first cassette consisted of the ligand-binding domain (LBD) of the *BmaEcR* fused to a heterologous GAL4 DNA binding domain. This fusion ORF was placed under the control of the CMV intermediate early promoter and terminated with an Sv40 poly-A addition signal. The second cassette contained the open reading frame (ORF) for the human RXR chimera fused to the VP16 activation domain. This chimeric ORF was placed under the control of the SV40 promoter and terminated with the bovine growth hormone poly-A addition signal. Finally, a reporter *Gaussia princeps* secreted luciferase reporter ORF was created that was preceded by five copies of the GAL4 response element and a minimal promoter. The reporter ORF was terminated with the human B globin poly-A addition signal. All cassettes were prepared using standard molecular cloning procedures and amplified by PCR with appropriate primers to produce products ready for recombination cloning. The three cassettes were then cloned by recombination into three pDONR vectors of the multisite Gateway Pro system (S1 Fig). The sequence of the resulting entry clones were confirmed and the functionality of the entry clones confirmed by transiently transfecting all three pDONR plasmids into HEK293 cells and testing for reporter activity in cells cultured in the presence and absence of 20E, as described below. The entry vectors were then inserted by three-way recombination into the destination vector pJTI Fast Dest (Invitrogen, CA) to create a destination vector containing all three cassettes (S1 Fig). This construct was validated by DNA sequencing and by functional assays, as described above. The destination vector was then used to create a stable mammalian cell line in HEK293 cells, using the Jump In Fast Gateway Targeted Integration System (Invitrogen, CA). Clones were selected in the presence of 30ug/mL hygromycin following the manufacturer’s protocol, and individual clones selected and screened for reporter expression in the presence and absence of 20E, in order to identify the clone producing the greatest signal to noise ratio and minimal well-to-well variation. The clone that exhibited highest signal to noise ratio was cultured and maintained in T75 flasks using 1x MEM media fortified with 10% FBS.

### Screening strategy and screening of ecdysone agonists

An ecdysone analog library consisting of compounds found to be active against various insect species was tested against the *Bma*EcR [26–28]. Thirty-five thousand cells of the cell line developed as described above were seeded into each well of a 96 well tissue culture plate. The cells were grown under the same conditions as described above. Cells were incubated at 37°C for 18–24 hours until they reached a density of 70–90%. Compounds were added to wells containing the cells at a concentration of 10μM and the cells incubated in the presence of the compounds for 48 hours. Control wells received ethanol, the vehicle of 20E. A total of 10μl of the culture media was removed from each well, and assayed for the presence of secreted luciferase using the renilla assay reagent in the Dual Luciferase assay kit (Promega, WI) following the manufacturer’s protocol. Each compound was assayed in triplicate. Student’s t-test was used to calculate the significance of any agonist activity observed, and the Bonferroni correction was applied to the results to adjust for multiple comparisons. Tenfold dilutions of compounds that resulted in a statistically significant increase in reporter activity (p < 0.05, Bonferroni corrected) were then assayed in triplicate to calculate EC_50_ values. To assess cytotoxic effects of the compounds on the mammalian cell line, cell viability was assayed with Alamar Blue (Thermo-Fisher Scientific, MA) according to manufacturer’s protocol. Z’ values for the assay were calculated as previously described [29].

### Homology model construction, validation and refinement

The tertiary structure of the *Bma*EcR was predicted using the Schrödinger’s Prime 3.1 comparative homology modeling module (Prime. Schrodingers, LLC New York, NY version 3. ed. 2012) [30]. Due to the lack of an experimental crystal structure of the *Bma*EcR, a search for suitable template structures upon which to model the *Bma*EcR tertiary structure was undertaken using BLAST (Basic Local Alignment Search Tool) [31]. The hemipteran *Bemisia tabaci* EcR (PDB ID: 1Z5X) was predicted to be the template with highest homology to *Bma*EcR (BLAST-bit score: 145.2) with excellent residue conservation. Based on the template secondary structure, Clustalω [32] was used to optimize the placement of the *Bma*EcR (LBD) α-helices and β-sheets using a hidden Markov model (HMM). After the Clustalω alignment, backbone atoms for aligned regions and conserved residue side chain atoms were directly transferred from the template to the query sequence to create an initial structure, followed by adding insertions and closing gaps using a knowledge based approach. The *B*. *tabaci* EcR native substrate ponasterone A was retained in the binding site during the homology model construction. The prime serial loop sampling protocol was used to refine one particular loop of importance (residues 162–175) that constitutes an intrinsic part of the binding site. To alleviate possible steric clashes between residues after model construction, Truncated Newton Conjugate Gradient (TNCG) minimization was performed using implicit solvent (VSGB 2.0) with the OPLS-2005 force-fields, until convergence was reached (< 0.01 kcal.mol^-1^ Å^-1^) [33]. The quality of homology model was verified by generating Ramachandran plots and further compared to the homology model generated with the I-Tasser web-server (details in S1 Appendix) for additional structural validation purposes [34]. To further refine the homology model, Molecular Dynamics (MD) simulations were performed for 65 ns while monitoring structural properties such as radius of gyration (R_g_), root mean square deviation (RMSD), root mean square fluctuation (RMSF), and total energy of the system (details in S1 Appendix). A snapshot of the *Bma*EcR homology model that was most representative of the complete 65 ns simulation (i.e., a snapshot that likely details biologically relevant conformations) was selected according to a simulation-clustering scheme (S1 Appendix); this structure was used for subsequent molecular docking studies.

### Molecular docking studies

All ligand structures were prepared using Schrödinger’s LigPrep 2.3 [35] module to produce appropriate 3D structures with correct stereochemistry, protonation states, and ring conformations. All ligands were then virtually docked in the *Bma*EcR homology model using the Glide 5.8 docking program. Glide uses a user-defined grid to determine the shape and properties of the binding site. The *Bma*EcR grid was defined by selecting the centroid of ponasterone A with otherwise default settings. The ligands were first docked and scored according to Glide SP (standard precision) and then re-docked with the Glide XP (extra-precision) protocol. Glide Scores (GScores) were calculated to estimate the relative predicted binding affinity amongst the ligands for the EcR binding site [36]. As part of the default Glide docking procedure, the van der Waals radii of non-polar hydrogen atoms were scaled by a factor of 0.8 and all calculations were performed with the OPLS-2005 force field.

### Validation of *in-silico* screening model

To illustrate the utility of combining virtual screening results with experimental assays, we generated a small virtual compound library to identify potential novel *Bma*EcR LBD hits. This small library was assembled from searches done on extensive and diverse online databases (i.e., the PDB and PubChem). The initial search used the ProBiS-CHARMMing / ProBiS-Ligands structural bioinformatics tool (available at http://probis.nih.gov), which compares the binding site of a provided structure to all known protein binding sites in the non-redundant PDB (currently 42,270 in total) [37,38]. The *Bma*EcR homology model was submitted to ProBiS, with the EcR LBD identified as the binding site of interest. Four proteins were identified with similar binding sites to the EcR LBD and each of these binding sites had several associated ligands; all of these were added to our virtual library. Secondly, a ponasterone A structure similarity search was performed on the PubChem compound library (consisting of ~83 million compounds) [39]. Ultimately, 104 compounds were identified via the two search protocols and added to the virtual library.

This library was then virtually screened according to the following protocol: (1) compounds were docked in the EcR LBD with Glide SP, (2) structures that resulted in binding scores of -7.25 kcal/mol (the binding score of 20-hydroxyecdysone) were re-docked into the EcR LBD using Glide XP docking, and again (3) those structures with Glide XP docking scores of less than -7.25 kcal/mol were docked into the EcR LBD with a flexible ligand-flexible receptor docking method known as Induced Fit Docking (IFD) [40,41], which allows for better binding mode prediction, albeit at a higher computational cost [42–44].

Four of the compounds identified by the virtual screen (AM580, BMS493, 22-R-Hydroxycholesterol, TTNPB) were found to be readily commercially available. These were obtained from Sigma-Aldrich (St. Louis MO, USA), and tested for activity and cytotoxicity using the assays described above.

### *In-vitro* phenotypic studies comparing the effect of Muristerone A and Ponasterone A with 20E

Adult females were cultured in a 6-well plate (five parasites per well) in CF-RPMI media (RPMI 1640 supplemented with 25 mM HEPES buffer, 2 mM glutamine, 100 U/ml streptomycin, 100 μg/ml penicillin, 0.25 μg/ml of amphotericin B, and 10% heat-inactivated fetal calf serum). Two biological replicates were conducted per treatment group. Parasites were allowed to acclimatize for 24 hours before treatments commenced. After the acclimatization period, Muristerone A, Ponasterone A and 20E (separate wells) were added to a final concentration of 10 μM to the experimental wells. The control wells received ethanol (the vehicle for ponasterone A, muristerone A and 20E). The parasites were then cultured for an additional four days. The culture media was changed every 24 hours. Three 20ul aliquots from each biological replicate were removed every 24 hours from each well and the number of progeny present (aborted eggs and embryos, immature microfilariae and mature microfilariae) counted. Thus, three technical replicates were performed on each biological replicate at each time point.

## Results

**Fig 1:**
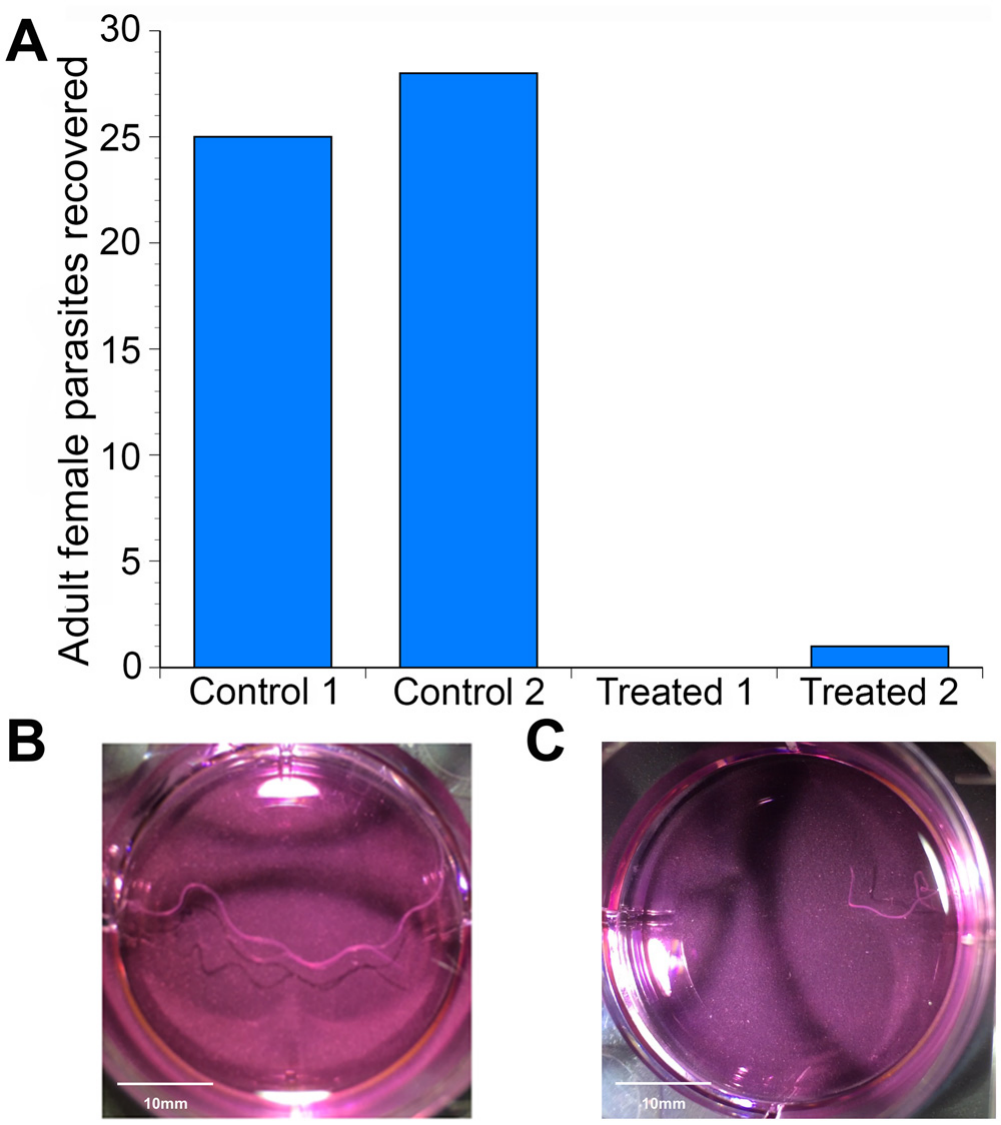
*In-vivo* phenotypic study demonstrating effects of 20-hydroxyecdysone on adult worms. **(A)** Graph showing recovered parasites from the study animals after necropsy. **(B)** Control worm showing normal length of the adult female parasite. **(C)** Ecdysone treated worm showing stunted growth and shorter length.

Previous studies have demonstrated that 20E stimulated expulsion of immature progeny (eggs, embryos and immature microfilariae) from adult female parasites cultured in the presence of 20E [45]. However, by analogy to its role in insects, the *Bma*EcR could be hypothesized to play an important role in molting in the filarial parasites as well. To test this hypothesis, the effect of 20E on the development of infective larvae to the adult stage was assessed in gerbils intraperitoneally infected with *B*. *malayi* infective larvae. Infected animals were given 20E at a dosage of 5mg/kg/day for a total of 150 days, the time necessary for infective larvae to develop into fertile adult parasites. After 150 days, the animals were euthanized and the peritoneal cavity was examined for parasites. A total of 25 and 28 adult female parasites respectively were recovered from the peritoneal cavity of the two control animals given only ethanol, the vehicle for 20E (Fig 1A). In contrast, no parasites were recovered from one of the animals given 20E, while a single incompletely developed female parasite was recovered from the second 20E treated animal (Fig 1B and 1C). In keeping with this observation, a large number of mature microfilariae were recovered from both control animals while no microfilariae were observed in the peritoneal cavity of the two treated animals.

**Fig 2:**
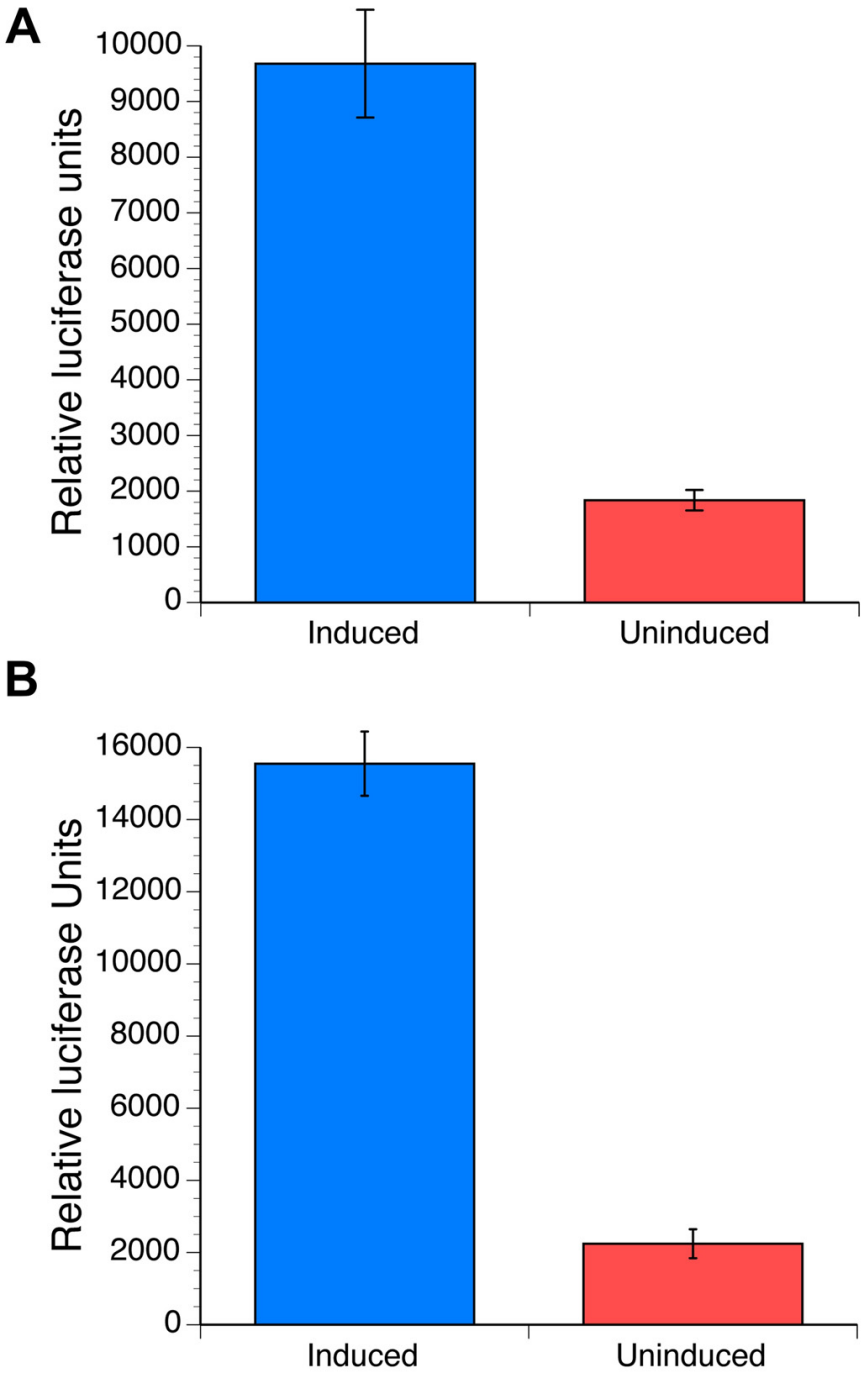
Performance of the mammalian cell assay. Results of a typical assay carried out in a 96 well format are shown. Columns indicate the mean and error bars the standard deviation of six control (uninduced) and six experimental (induced) wells. **(A)** Assay conducted with the transiently transfected cells with 150ng per well of each construct (*Bma*EcR- GAL4, RXR-VP16, GLuc reporter) using Lipofectamine, and treated with 10μM 20-hydroxyecdysone for 48 hrs. **(B)** Assay conducted with the stably transfected cells.

Previous studies have demonstrated that mammalian cells transiently transfected with three plasmids containing ORFs for the *Bma*EcR LBD domain, a heterodimeric RXR partner and a secreted luciferase reporter exhibited an up-regulation of reporter activity when cultured in the presence of ponasterone A, a potent ecdysone analog [13]. Mean luciferase activity in the media of HEK293 cells transiently transfected with these three plasmids increased roughly seven-fold when compared to the reporter activity in the media of cells cultured in the absence of 20E (Fig 2A). The Z’ calculated for this assay was 0.72, suggesting that an assay based upon such a platform might be suitable for use in a high throughput format to identify agonists or antagonists for the *Bma*EcR. However, the transient transfection process varied in efficiency from day to day and the need to repeatedly transiently transfect cells limited the throughput of the assay, both of which were impediments to adapting the assay to a high throughput format. To overcome these obstacles, a stable cell line was created that incorporated the three cassettes necessary for the assay (the *Bma*EcR LBD, the RXR partner and, the reporter) into the genome of HEK293 cells. Assays using the stably transfected cells revealed a performance comparable or better than that of the assay using the transiently transfected cells, with a signal to noise ratio ranging from 4.5 to 5.5 and a Z’ that ranged from 0.78 to 0.88 when conducted on different days (Fig 2B).

**Fig 3:**
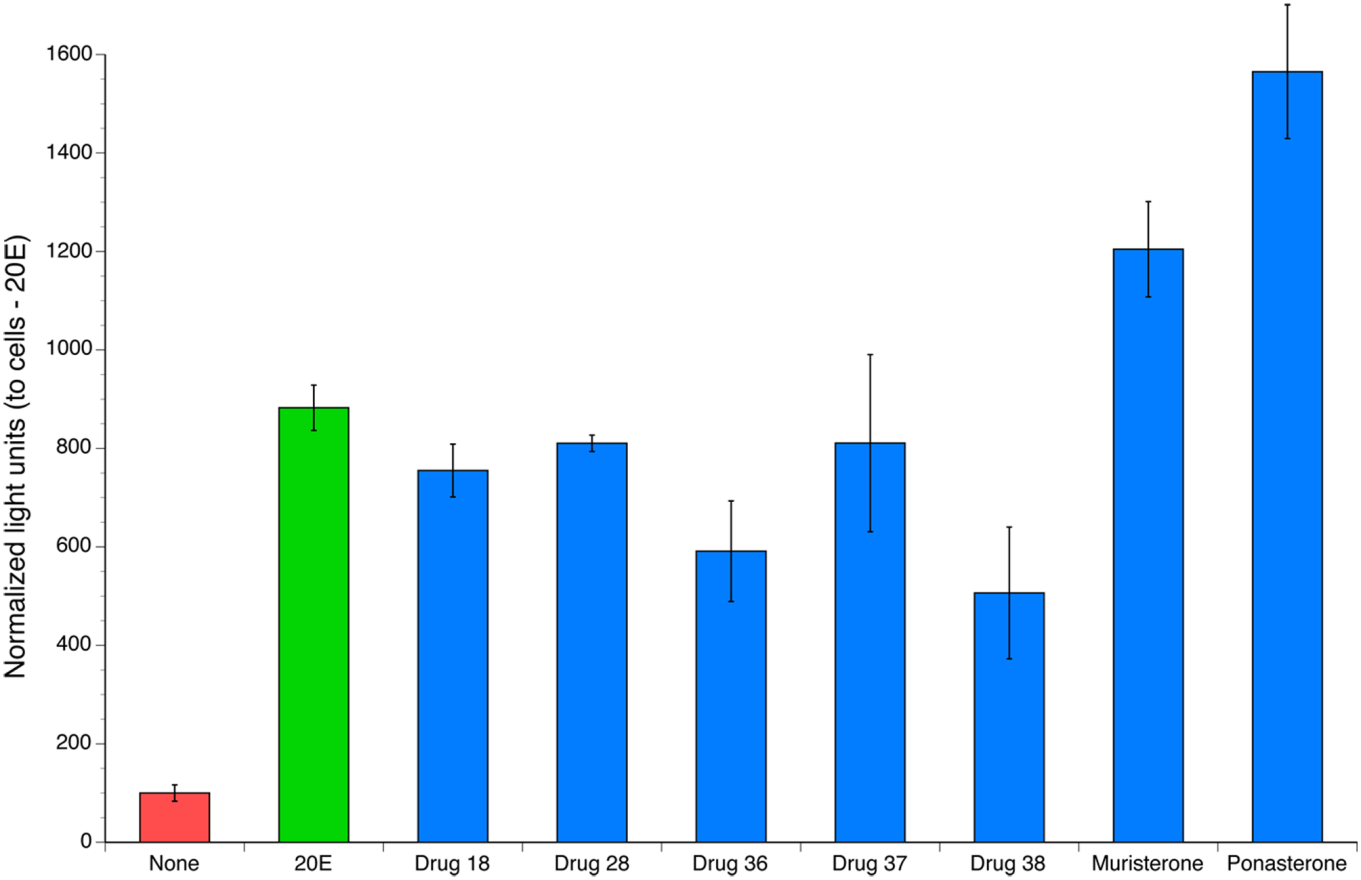
Compounds identified in the screen of compounds active against the *BmaEcR*. Cells were treated with 10μM of each drug for 48 hrs. The blue bars represent all other compounds tested; 20E is represented as green bar and the red bar indicates the activity present in untreated cells. All columns represent the mean and the error bars the standard deviation in triplicate wells. The horizontal line indicates the activity expected for compounds with no activity.

The stably transfected cells were used to screen a focused library comprised of 40 compounds belonging to the diacylhydrazine, tetrahydroxyquinoline and the steroidal ecdysone analog families. These compounds were tested for activity against *BmaEcR* using the screening strategy described in Materials and Methods. The initial screen identified seven compounds that resulted in a significant increase in reporter activity in the stable cells cultured as compared to the activity in the cells cultured in the presence of solvent alone (Fig 3, p < 0.05, with Bonferroni correction for multiple comparisons). In particular, muristerone A and ponasterone A demonstrated a higher level of activity than 20E in this assay.

**Fig 4:**
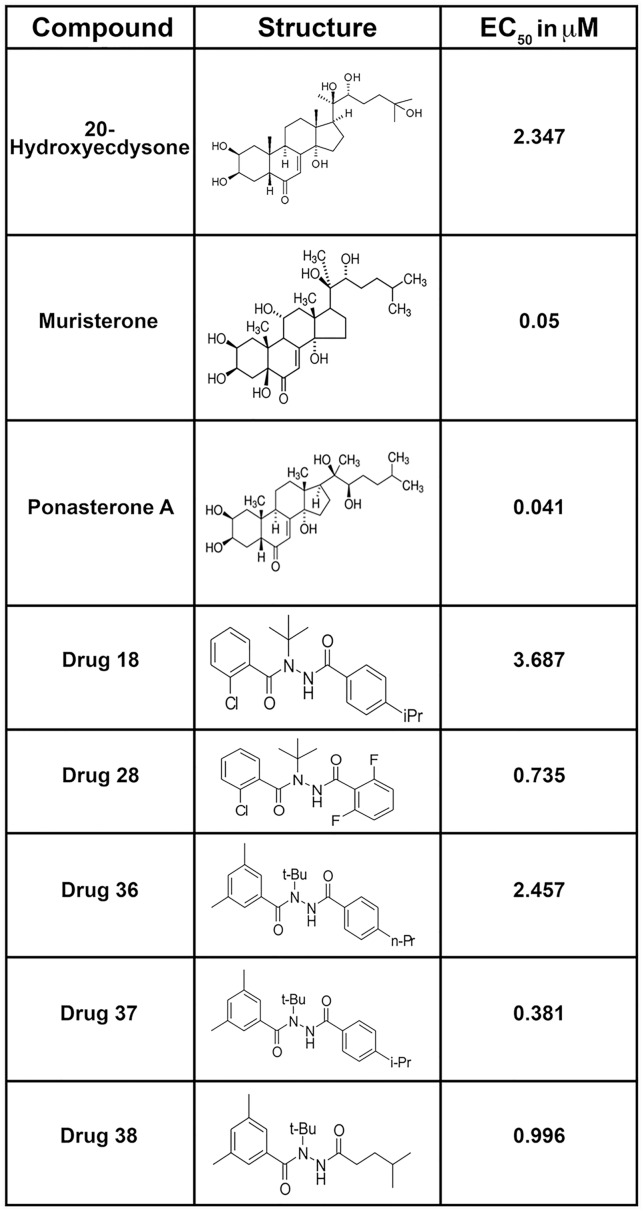
EC_50_ values and structures of compounds identified in the preliminary screen of compounds interacting with the *Bma*EcR using the cell-based luciferase assay.

The compounds identified as agonists were then tested at a series of concentrations to determine the EC_50_ values. The EC_50_ values for the initial hits were low, with some exhibiting values that were in the sub-μM range (Fig 4). None of the compounds exhibited cytotoxicity at a concentration of 10 μM.

**Fig 5:**
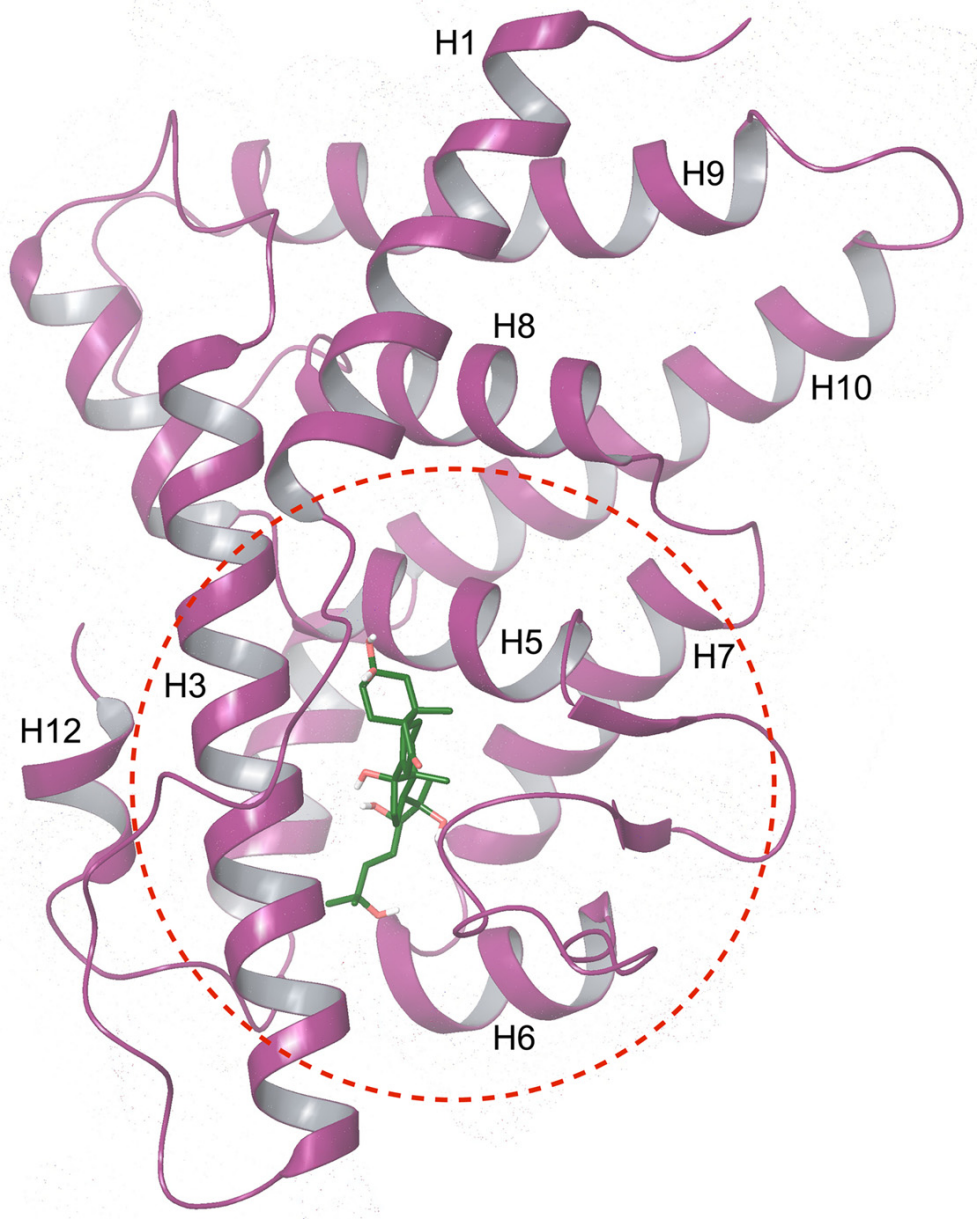
Cartoon depiction of the *B*. *malayi* EcR LBD homology model. The secondary structural element are colored in purple. The red dotted circle denotes the active site for the docking studies.

A homology model of the *Bma*EcR LBD was then constructed as described in Materials and Methods. The Ramachandaran plots before and after MD simulations were created to discern potential changes in ϕ /ψ distributions. The number of residues within the favored and allowed regions increased from 89.6% to 98.7% as a result of MD simulations (S2 Fig). Fig 5 presents the structure of the *B*. *malayi* EcR LBD homology model with Ponasterone A occupying the active site.

**Fig 6:**
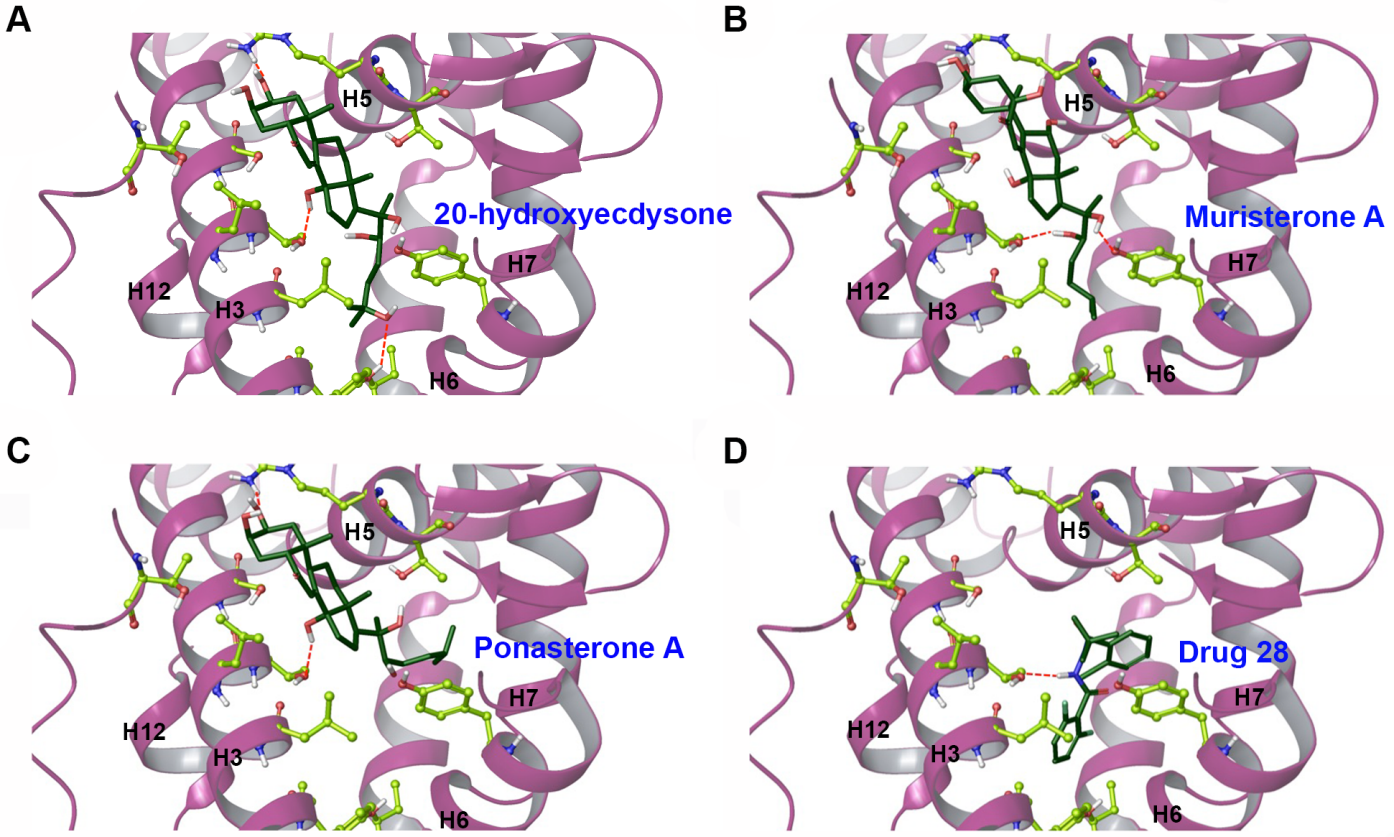
Predicted structures of agonists docking in the active site of the homology model of the *Bma*EcR LBD. **(A-D)** The panels show 20-hydroxyecdysone, ponasterone A, muristerone A and diacylhydrazine #28 binding into the active site.

The homology model was used for virtual screening studies of agonists identified with the molecular assay above to verify the model performance. The structure with the most favorable protein—ligand interaction energy based on the MD simulation (according to an RMSD clustering scheme of the final 20 ns) was chosen as the representative structure for molecular docking studies (details in S1 Appendix). The 3D structures of the ligands were docked and scored using both Glide SP (standard precision) and Glide XP (extra precision) (Fig 6). The predicted lowest energy for each agonist (ligand) is described in detail in the S1 Appendix and S2 Table. A comparison of computational and experimental results is shown in Table 1. A Pearson correlation coefficient of 0.78 was found between experimental log EC_50_ values and Glide XP docking scores, suggesting that the model predictions trended well with the data collected from the mammalian cell assay.

**Table 1:** XP descriptor analysis given from XP docking of hormones and agonists in *B*. *malayi* EcR-LBD binding site.

Compound	EC_50_ μM	Log EC_50_	GScore
20-Hydroxyecdysone	2.34	0.37	-7.25
Muristerone A	0.05	-1.3	-8.28
Ponasterone A	0.04	-1.39	-9.37
Diacylhydrazine # 28	0.74	-0.137	-8.61
Diacylhydrazine # 37	0.38	-0.42	-7.50
Diacylhydrazine # 18	3.69	0.57	-7.37
Diacylhydrazine # 38	0.99	-0.001	-7.27
Diacylhydrazine # 36	2.46	0.397	-6.59

**Fig 7:**
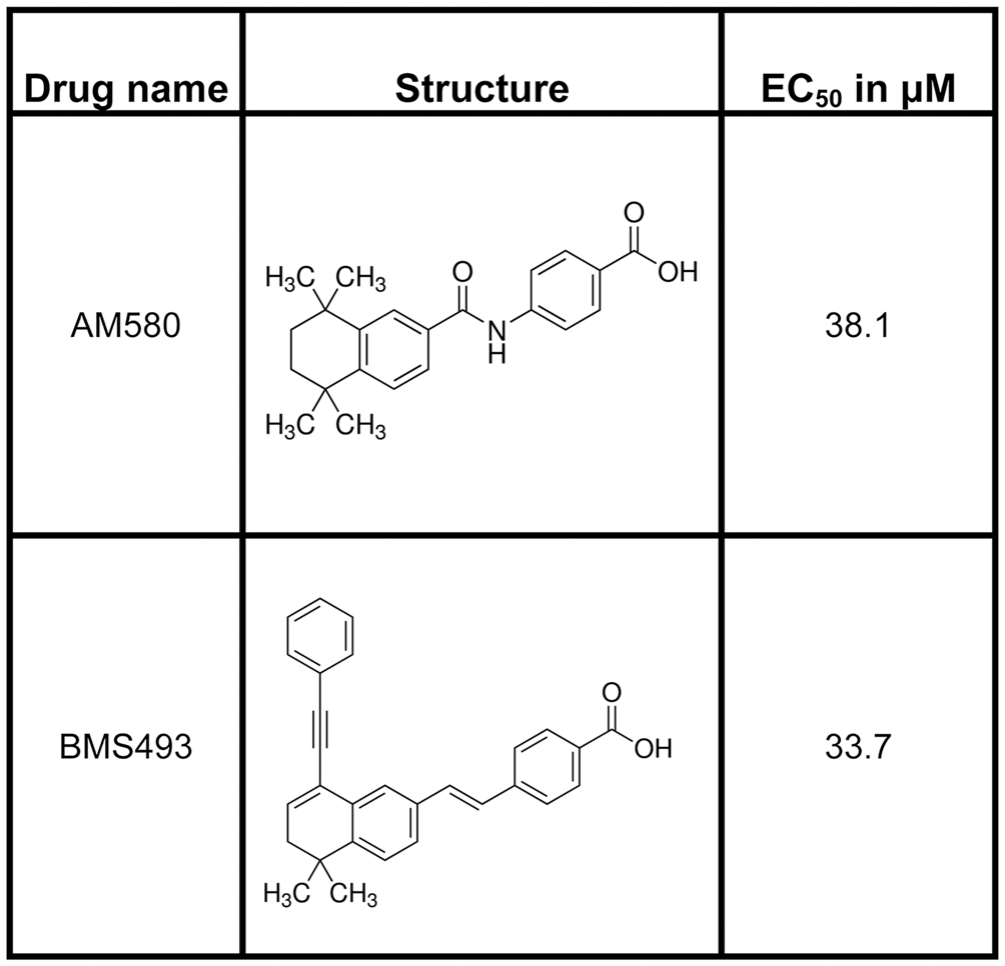
Structure and EC_50_ values of the compounds predicted to be *Bma*EcR ligands using the *in-silico* virtual screening method.

As a first test of the utility of the model to identify potential compounds capable of interacting with the BmaEcR, a number of databases were initially screened as described in Material and Methods, resulting in a library of 104 potential hits. A total of 25 compounds survived the selection protocol described in Materials and Methods (i.e. had Glide XP predicted binding scores more negative than -7.25 kcal/mol). The binding modes of these 25 compounds were further refined via flexible ligand—flexible protein docking (i.e., IFD). The docking scores for these 25 compounds are shown in S3 Table. Of particular note, two compounds found to be active were isolated without using the ligand’s structural information (i.e., were obtained via ProBis CHARMMing / ProBis Ligands). Out of these 25 compounds, four (AM580, BMS493, 22-R-Hydroxycholesterol, TTNPB) were commercially available. When tested on the cell-based assay, AM580 and BMS493 were active agonists with micromolar EC_50_ values (Fig 7). The remaining two compounds (22-R-Hydroxycholesterol and TTNPB) were found to be cytotoxic at all concentrations tested, and could not be evaluated in the cell based assay.

**Fig 8:**
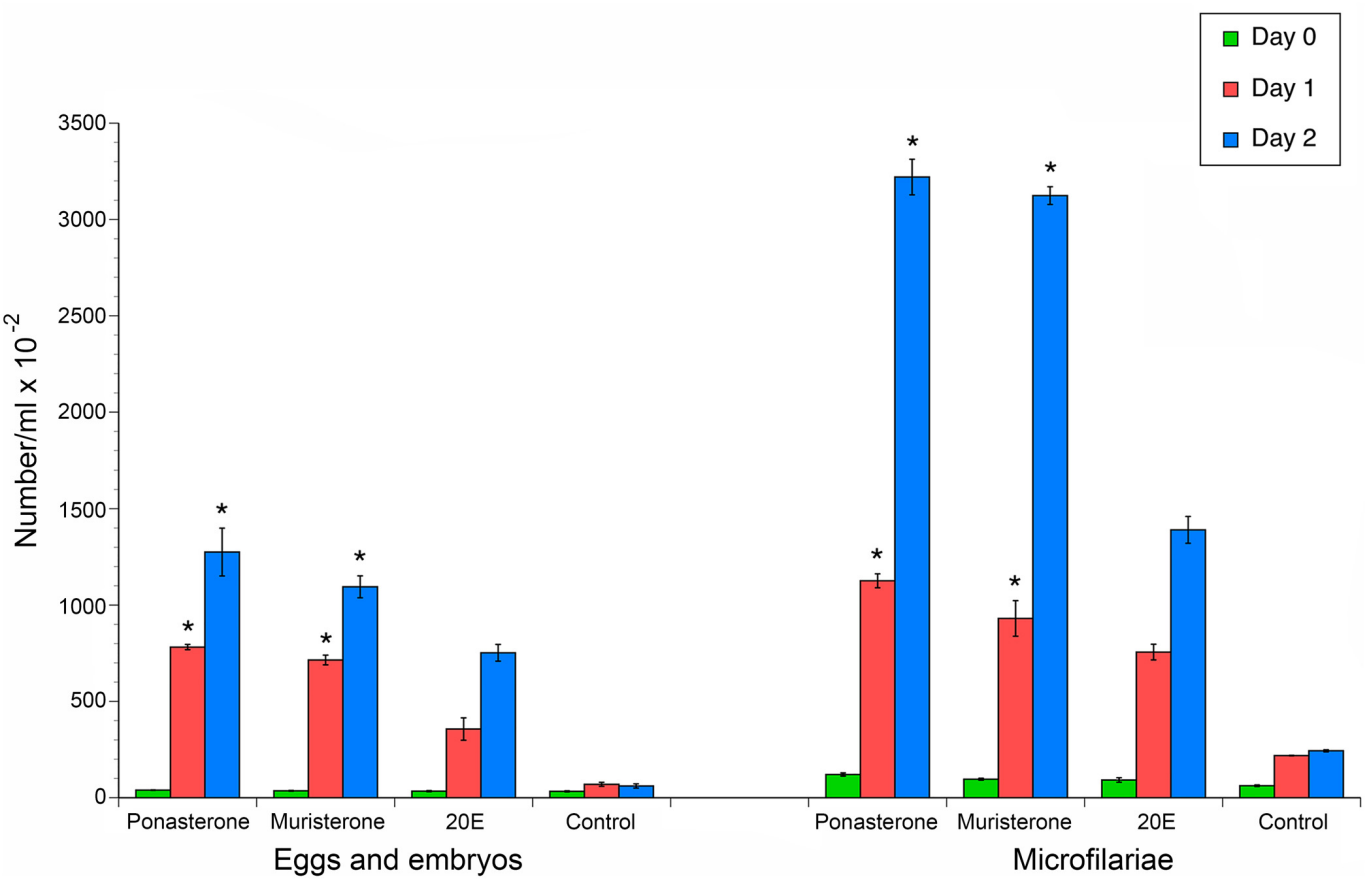
*In-vitro* phenotypic study demonstrating effect of Ponasterone A, Muristerone A and 20E on adult female worms. The bars depict counts of progeny expelled per ml of media. 1 and 2 represent two biological replicates of the treated and control for the expulsed progeny. The error bars denote the standard deviation of the technical replicates. The colored bars represent each consecutive day of the experiment.

As mentioned above, previous studies have demonstrated that culturing gravid adult females in the presence of 20E induced expulsion of immature eggs, embryos and microfilaria. Ponasterone A and muristerone A were the compounds that gave a better performance than 20E in the cell assay as well as having a high predicted binding score in the active site of the *BmaEcR* homology model. Thus, it was of interest to determine if these compounds could elicit a similar response. As predicted, gravid adult female worms cultured in presence of 10 μM muristerone A and ponasterone A exhibited a significant increase (p<0.001, t-test) in expulsion of aborted eggs, embryos and microfilariae as compared to the parasites cultured in the presence of the 20E (Fig 8).

## Discussion

*Brugia malayi* is an ecdysozoan that exhibits molting and developmental changes during the lifecycle similar to those seen in arthropods. Studies have shown a high expression of the *Bma*EcR transcripts in *B*. *malayi* in adult females, L3, eggs and embryos and mature microfilariae [23]. In keeping with this expression pattern, recent studies have demonstrated that 20E affected egg development and embryognesis in adult female *B*. *malayi* [45]. The data presented above, though limited, suggests that 20E also interferes with the development of infective larvae to adult parasites, a process involving two molts. In these studies, animals infected with L3, when treated with 20E for 150 days (the time necessary for the parasites to develop from L3 to fecund adult parasites) exhibited a dramatic decrease in the number of adult worms recovered after treatment, along with a complete inhibition of microfilarial production. While the experiment was designed to look for an effect at any point during this prolonged devellopmental process, it is likely that 20E was actually interfering with some temporally limited critical developmental processes ocurring during this period, such as the L3-L4 molt or the molt from L4 to immature adult parasites. Thus, treatment throughout the entire 150 day period may have not been necessary. In support of this hypothesis, L3-stage larvae cultured *in-vitro* in the presence of 20E molt earlier (on day 7–8) when compared with larvae cultured in the absence of 20E, which between day 9–11 (S4 Table). It is therefore possible that administration of 20E results in inappropriate activation of the *Bma*EcR, resulting in premature molting and death of the developing parasites *in vivo*. This result, when combined with the effect of 20E on cultured adult females, suggests that 20E has effects on both embryogenesis and development of *B*. *malayi* from the infective larvae stage to the adult stage. This finding would be similar to the role that 20E plays in insects, where is serves as a master regulator of both vitellogenesis and molting [46]. Further, these observations suggest that agonists of 20E may represent an important potential chemotherapeutic space for the development of novel drugs against the human filaria.

Previous studies demonstrated that mammalian cells transfected with plasmids encoding three individual ORFs could be used to detect activation of the *Bma*EcR [13]. The initial assay utilized NIH3T3 cells, which grow slowly and are therefore not optimal for use in a high throughput assay format, where large quantities of cells are required. For this reason, we decided to attempt to utilize HEK293 cells, which grow more quickly and therefore would be more useful for high throughput assays. The assay, when optimized with HEK293 cells, demonstrated an improved signal to noise ratio and Z’ value when compared to the transient transfection assay utilizing NIH3T3 cells (S5 Table). However, because the assay relied on transiently transfected cells, it was cumbersome and subject to day to day variations. For this reason, we developed a cell line into which all three of the necessary cassettes were inserted into the host cell’s genome. This assay exhibited performance characteristics that equaled or exceeded those of the original assay, making it much more amenable to a high throughput format.

As a first test of the assay, a small library of compounds that have previously been shown to act as ligands for EcRs of other species, as well as ligands for other members of the NHR family were screened for activity aginst the *Bma*EcR. The results of this study suggested that members of the diacylhydrazine (DAH) family were active ligands for the *Bma*EcR. This class of compounds has been previously exploited by the agricultural industry to develop insecticides targeting insect EcRs. The DAHs cause precocious molting, leading to the death of the the target organism. These compounds also show high species specificity. Furthermore, there is no evidence of detrimental effects of DAH on the vertebrates. Taken together, these data suggest that members of the DAH family may have good potential as chemotherapeutic agents against the filaria.

As described above, ecdysone analogs (both steroidal and non-steroidal) often demonstrate species-specific activities. In the studies reported here, we see similar evidence of species-specific activities for compounds active against the *Bma*EcR. For example, muristerone A, a known ecdysone analog, was one of the compounds in the library tested with the *Bma*EcR cell based assay. Muristerone A has demonstrated activity in the insect system [47], but it did act as an agonist when tested against the *Bma*EcR.

Apart from the DAH family, several other families of compounds, including the tetrahydroquinolones, butyl-benzamide and acylaminoketone, have been shown to act as ecdysone agonists. Other potential sources of chemotherapeutic agents may include compounds showing activity against other members of the NHR family; e.g., natural extracts from plants and fungi. The development of the assay based upon the stable cell line described above should be useful in implementing high-throughput screens to identify potential additional leads from these sources.

To complement the high throughput assay, a homology model was also created to permit *in-silico* screening of potential ligands against the *Bma*EcR. When using homology models to predict ligand binding, it is important to verify whether the resulting models are: a) likely to be found in nature, b) structurally relatable to other proteins in their family (i.e., have the same overall fold), and c) are reproducible. Analysis presented via Ramachandaran plots and the direct comparison to known protein relatives together suggests that the homology model reported above meets these critera and that it closely represents the true structure of the *Bma*EcR LBD. Furthermore, the model reflects the known mechanism of binding of the EcR to its cognate ligand, in that upon binding of 20-hydroxyecdysone, a conformational change allows H12 to interact with the ecdysone response elements present in the promoters of ecdysone responsive genes, activating transcription. The fact that the model captures this mechanistic feature provides added support for its potential utility in virtual screening studies.

In the analysis of predicted binding poses for each ligand, a pattern was observed: orientation of the potential steroidal ligands were optimized such that hydrophobic tails were buried in a hydrophobic region, while the central steroid nucleus structure was surrounded by more hydrogen bond donating/accepting side-chains. When the agonists identified in our preliminary screen were docked in the *Bma*EcR LDB, they interacted almost exclusively with the hydrophobic region, resulting in very favorable hydrophobic enclosure and lipophilic terms (as listed in XP energy decomposition analyses, Table 1). The DAH family occupied a very different orientation in the active site when compared to the steroidal ligands, yet displayed comparable activity to stimulate the receptor.

Ponasterone A and muristerone A displayed a higher fold change activity than 20E in the stable cell line assay as well as being predicted to bind firmly in the active site of the *Bma*EcR homology model. In confirmation of this, ponasterone A and muristerone A increased expulsion of microfilariae and aborted eggs and embryos from adult female parasites cultured in their presence. This short-term culture assay can therefore be of use in providing a preliminary confirmation of the biological activity of potential leads identified in the high throughput and *in-silico* screens prior to undertaking time consuming and expensive *in-vivo* studies in infected animals.

In conclusion, the data presented here validate the filarial ecdysone receptor as a potential chemotherapeutic target, and describe a series of assays that can be used to identify and validate potential lead compounds against this target. Given the success of the agricultural industry in targeting this receptor, these studies lay the groundwork for the exploitation of this receptor to develop anti-filarial agents that could supplement those currently used in the elimination programs worldwide.

## Supporting Information

S1 Appendix Homology modeling and virtual screening studies. (DOCX)

S1 Fig Strategy for production of a stable mammalian cell line for screening for agonists and antagonists of the *Bma*EcR. ‘X” = Gateway recombination cloning. Colored circles schematically indicate the specific sequences used in the Gateway recombination cloning reactions.
(TIF)

S2 Fig Ramachandaran plot compiled of the phi-psi backbone dihedrals from the model after molecular dynamics simulations. The legend illustrates the color designations of certain regions in the plot. The sum of the first two percentages indicates the number of residues in more favored regions (98.7%).
(TIF)

S3 Fig Superposition of Prime homology model (yellow) over I-Tasser homology model (blue). (TIF)

S4 Fig MD simulation results. A) RMSD plotted over the course of 65 ns simulation, B) radius of gyration plotted over the course of 65 ns simulation, C) total energy of the homology model and ponasterone A complex monitored over the course of 65 ns simulation, and D) RMSF analysis for each residue in the EcR LBD homology model.
(TIF)

S5 Fig Predicted structures of compounds docked in the active site of the *Bma*EcR LBD. Panel A: Compound 37. Panel B: Compound 38. Panel C: Compound 18. Panel D: Compound 36.
(TIF)

S1 Table Summary of RMSFs calculated from the MD simulation of for all residues within 3 Å of the 20-hydroxyecdysone binding site. Residue name is given on the left of each column and the RMSF is given to on the right. Residues are ordered by their residue number. Residues constituting the “hydrophobic pocket” are underlined.
(DOCX)

S2 Table Summary of XP energy decomposition analyses. (DOCX)

S3 Table Compounds identified by the *in-silico* virtual screening and their docking scores. (DOCX)

S4 Table Molting of L3 stage larvae when treated with 20-hydroxyecdysone. (DOCX)

S5 Table Comparison of the transient transfection assay when conducted with NIH3T3 and HEK293 cells. (DOCX)
