# Supplementary material for: Identification of Ecdysone Hormone Receptor Agonists as a Therapeutic Approach for Treating Filarial Infections
Source: PLoS Negl Trop Dis. 2016 Jun 14;10(6):e0004772. doi: 10.1371/journal.pntd.0004772 (PMC4907521; doi:10.1371/journal.pntd.0004772)
Supplement: S1 Appendix — (DOCX) [file pntd.0004772.s001.docx]

**S1 Appendix: Homology modeling and virtual screening studies**

**Homology model construction and validation**

A Ramachandaran plot was compiled using PROCHECK [1] to compare the distribution of backbone dihedrals (φ/ψ) of our EcR homology model to the known distributions of these dihedrals amongst all crystallized proteins in the Protein Data Bank. 89.6% of all amino acids in our homology model had phi-psi dihedral pairs plotted within “favored” regions of backbone conformations, i.e., regions that are highly populated by amino-acids in “nature-made” polypeptides or proteins. This result supports that our constructed homology model has biologically relevant protein conformation with regards to backbone orientation which relates to minimization of backbone strain. To verify the reproducibility of our homology model, the online wed-server, I-TASSER, which constructs homology models from submitted sequences, was used to construct a second homology model of the *Bma*EcR-LBD. As is shown in S3 Fig., our model and the top ranked I-TASSER [2] generated model agree on placement of secondary structural elements and the overall EcR fold is present in both. Furthermore, the two structures agree on placement of residues within the binding site.

Ecdysone receptors from *Bemisa tabaci* and *Heliothis vericens* insect species have been crystalized and described earlier [3]. As proteins with the same functions as, and closely related to the *B. malayi* EcR, these structures served as guides for comparision to the predicted tertiary elements of our *B. malayi* EcR homology model. Our model exhibits 10 of the 11 expected α-helices and the one expected β-sheet comprised of three strands, when compared to *B. tabaci* and *H. vericens* EcR crystal structures. The model also exhibits the same general fold common to the EcR family: 11 to 12 α-helices arranged antiparallel with respect to their neighbors and stacked in three “layers” with one β-sheet encompassing one side of the binding pocket. Additionally, our homology modeling protocol was able to predict the placement of α-helix-12 (H12), a secondary structural element that is crucial to the function of the EcR.

**Homology model refinement**

To refine the homology model, (i.e., allowing it to access more relaxed/biologically relevant conformations) and to ensure the model was stable, molecular dynamics (MD) simulations were carried out with the CHARMM (CHemistry at HARvard Molecular Modeling) biomolecular simulation program [4]. CHARMMing [5] and pdbtools [6] were used to convert the homology model to a CHARMM compatible version and to generate necessary topology and coordinate files. The protein was treated with CHARMM (C36) force-fields and parameters for substrate ponasterone A were generated using the CHARMM generalized force-field (CGenFF) via the Paramchem Web-interface [7]. A total of 76,128 TIP3 water molecules were added to solvate the homology model, and waters that overlapped with protein atoms were deleted. The resulting system was a cubic box of 80 x 80 x 80 Å with the EcR model at the center. Next, the system was neutralized by adding sodium and chlorine ions sequentially, replacing random water molecules, until the total salt concentration was 0.15 M NaCl. This neutralization step was followed by short (10 steps) of steepest descent (SD) minimization and then 25 steps of Adopted Basis Newton-Raphson *(*ABNR*)* minimization to alleviate steric clashes that may have arisen during the ion placement. Slowly the system was heated from 100 K to 300 K for 100 ps and after heating the system was equilibrated and simulated for a period of 65 ns under isothermal-isobaric conditions (NPT ensemble). A 2 fs integration time step was used, and snapshots were saved for every 1000 steps (2 ps). The SHAKE algorithm constrained covalent bonds to hydrogen atoms. Particle mesh Ewald (PME) was used for evaluating long-range electrostatics and a 12Å cut-off was used to calculate LJ-nonbonded terms. To improve computational speed, the 65ns simulation was conducted with GPU accelerate code and the CHARMM/OpenMM interface [8]. To monitor the structural stability and convergence of the homology model across the simulation, structural properties like radius of gyration (R_g_), root mean square deviation (RMSD), root mean square fluctuation (RMSF), and total energy of the system were reported. These time correlated properties are shown in S4 Fig. panel A, B and C. Additionally, root mean square fluctuation (RMSF) was calculated for each residue (using the complete 65ns trajectory) to estimate which regions of the EcR model are most or least flexible (S4 Fig. panel D). RMSD and R_g_ plots support that the model was equilibrated within the first 20 ns of simulation. Total energy also remains stable, fluctuating steadily around -140500 kcal.mol^-1^; with no large total energy drifting indicated in S4 Fig. panel C, thus it is apparent that the simulation is converged. RMSFs are given in S4 Fig. panel D and S1 Table for those residues within 3Å of the hormone-binding site. As most residues presented in S1 Table exhibit RMSFs of < 2Å, indicating that binding site residues are relatively inflexible during MD simulations. Following the MD simulations, Ramachandran plots were regenerated and we have seen an increase in the population of residues just within the most favored regions of the φ-ψ distributions from 89.6 % to 90.5 % (Fig. S2).

**Clustering Scheme and Virtual Screening Studies:**

In order to obtain a single snapshot that represented the equilibrated portion of the 65 ns trajectory, all frames from the last 20 ns were clustered according to an RMSD radius cut-off of 0.50 Å using CHARMM’s CORREL module, this resulted in 10 unique clusters and these clusters were ranked based on population. RMSD calculations were performed by selecting all the residues that are within 5 Å of the ponasterone A substrate. Then, the interaction energy between ponasterone A and the protein system was calculated for each frame in the highest populated cluster. The frame with the lowest (most favorable) interaction energy was chosen as the representative structure of *B. malayi* EcR, and this structure was used to perform molecular docking studies.

The lowest energy pose (as predicted by Glide Score) of **20-hydroxyecdysone** bound in *B. malayi* EcR predicts the hormone’s hydrophobic tail to be stabilized via van der Waals interactions to several residues including Leu44, Ile120, Phe119, Tyr41, Ile222, and Ile220. However, the central gonane structure of 20-hydroxecdysone, is surrounded by residues in the binding pocket of polar character including Thr89, Thr48, Tyr116 as well as some hydrophobic residues, Leu47, Leu85, and Val100. GlideXP docking predicts hydrogen bonding between 20-hydroxyecdysone and residues Arg88, Thr48 and Tyr41. **Muristerone A** is oriented similarly to 20-hydroxyecdysone and ecdysone in the EcR LBD binding pocket with the hydrophobic tail interacting heavily via van der Waal and hydrophobic contacts to Ile120, Phe119, Tyr41, and Leu44. Muristerone A also participates in hydrogen bonding, donating hydrogen bonds to Tyr116-OH, Thr48-OH and Ser51-OH. **Ponasterone A** is also oriented in the binding site with the hydrophobic tail interacting with the grouped hydrophobic residues on one side, and the more polar end of the molecule interacting via hydrogen bonding. Leu85, Leu120, Phe128, and Met127 interact via van der Waals contacts with the tail and similar to 20-hydroxyecdysone and ecdysone, Arg88-NH_2_ donates a hydrogen bond to C_3_-OH of ponasterone A. Further, donates hydrogen bonds to Thr48 and Thr89.

Out of all agonists, compound 28 has the lowest Glide Score. **Compound “28”** is predicted to bind in the hydrophobic region of the binding pocket; out of the 17 residues within 5Å of compound 28, 16 are hydrophobic (within 3Å of 28: Ile120, Met127, Thr48, Leu85, Leu44, Val100, and Tyr116, within 5Å of 28: Leu47, Phe128, Leu123, Ile222, Tyr41, Phe119, Val86, Ile215, Ile220, Ala131, Leu102). GlideXP predicts compound 28, with two aromatic rings, participates in aromatic interactions to Tyr41 and Tyr116, both of parallel displaced style aromatic interaction. Additionally, much of the binding energy should be attributed to van der Waals interactions between alkyl substituents on the ligand and hydrophobic residues in the binding site including Val100, Met 127, Leu85, and Leu44. The Glide docking score Table 1 predicts compound 28 binds most favorably out of six agonists with GScore of -8.61 kcal.mol^-1^, and this is largely due to the highly favorable hydrophobic enclosure term of -2.00 kcal.mol^-1^ and the lipophilic term of -5.00 kcal.mol^-1^.

Like 28, **Compound “37”** binds in the hydrophobic binding pocket and is supported by van der Waals interactions to Ile120, Phe128, Ala124, Tyr116, and Leu85 (S5 Fig. panel A). Compound 37 also donates a weak (as the angle is not optimal) hydrogen bond to Thr89-OH. The calculated GScore for 37 is -7.50 kcal.mol^-1^, with largest contributing terms again being the hydrophobic enclosure (-1.30 kcal.mol^-1^) and the lipophilic term (-5.35 kcal.mol^-1^).

**Compound “18”** is predicted to also bind in the hydrophobic pocket and interact mostly via van der Waals terms to Ile120, Leu102, Leu44, Ile222, and Ile215 (S5 Fig panel B). Compound 18 is also predicted, like 28, to form parallel-displaced aromatic interactions to Tyr116 and to Phe128. The calculated GScore is -7.37 kcal.mol^-1^ for 18, with the highest contributions again from the hydrophobic enclosure term (-0.97 kcal.mol^-1^) and the lipophilic term (-5.30 kcal.mol^-1^).

**Compound “38”** binds in the hydrophobic pocket and interacts via van der Waals terms to Tyr116, Ile120, Ile120, Leu44, Phe128, and Leu47 (S5 Fig panel C). Additionally, Tyr116 forms a parallel-displaced aromatic interaction to compound 38’s aromatic ring and Thr48 accepts a hydrogen bond from compound 38’s-NH. Compound 38 has a GScore of -7.27, and again with the most prominent terms being the hydrophobic enclosure term (-0.65 kcal.mol^-1^) and the lipophilic term (-4.87 kcal.mol^-1^).

Finally **Compound “36”** binds in the hydrophobic binding pocket, and similarly interacts with Leu102, Val100, Phe128, Leu85, Ile120, and Ala124 via van der Waals contacts (S5 Fig panel D). GlideXP also predicts compound 36 will donate a hydrogen bond, from a nitrogen hydrogen, to Thr89-OH. Compound 36 has a GScore of -6.59 kcal.mol^-1^ with the hydrophobic enclosure term contributing -0.70 kcal.mol^-1^ and the lipophilic term contributing -4.65 kcal.mol^-1^.

**References**

1. Laskowski RA, MacArthur MW, Moss DS, Thornton JM. PROCHECK: a program to check the stereochemical quality of protein structures. Journal of Applied Crystallography. 1993;26(2):283-91. doi:10.1107/S0021889892009944.

2. Zhang Y. I-TASSER server for protein 3D structure prediction. BMC bioinformatics. 2008;9:40. Epub 2008/01/25. doi: 10.1186/1471-2105-9-40.

3. Carmichael JA, Lawrence MC, Graham LD, Pilling PA, Epa VC, Noyce L, et al. The X-ray structure of a hemipteran ecdysone receptor ligand-binding domain: comparison with a lepidopteran ecdysone receptor ligand-binding domain and implications for insecticide design. Journal of Biological Chemistry. 2005;280(23):22258-69. doi: 10.1074/jbc.M500661200.

4. Brooks BR, Brooks CL, Mackerell AD, Nilsson L, Petrella RJ, Roux B, et al. CHARMM: the biomolecular simulation program. Journal of Computational Chemistry. 2009;30(10):1545-614. doi: 10.1002/jcc.21287.

5. Miller BT, Singh RP, Klauda JB, Hodoscek M, Brooks BR, Woodcock HL, 3rd. CHARMMing: a new, flexible web portal for CHARMM. Journal of Applied Crystallography. 2008;48(9):1920-9. Epub 2008/08/14. doi: 10.1021/ci800133b.

6. Rodrigues J. PDBTools. Pdb-tools: First Stable Release2015.

7. Vanommeslaeghe K, Raman EP, MacKerell AD. Automation of the CHARMM General Force Field (CGenFF) II: assignment of bonded parameters and partial atomic charges. J Chem Inf Model. 2012;52(Copyright (C) 2015 American Chemical Society (ACS). All Rights Reserved.):3155-68. doi: 10.1021/ci3003649.

8. Hynninen AP, Crowley MF. New faster CHARMM molecular dynamics engine. Journal of Computational Chemistry. 2014;35(5):406-13. doi: 10.1002/jcc.23501. PubMed PMID: 24302199; PubMed Central PMCID: PMC3966901.
