# Supplementary figures and images for: Identification of Ecdysone Hormone Receptor Agonists as a Therapeutic Approach for Treating Filarial Infections
Source: PLoS Negl Trop Dis. 2016 Jun 14;10(6):e0004772. doi: 10.1371/journal.pntd.0004772 (PMC4907521; doi:10.1371/journal.pntd.0004772)

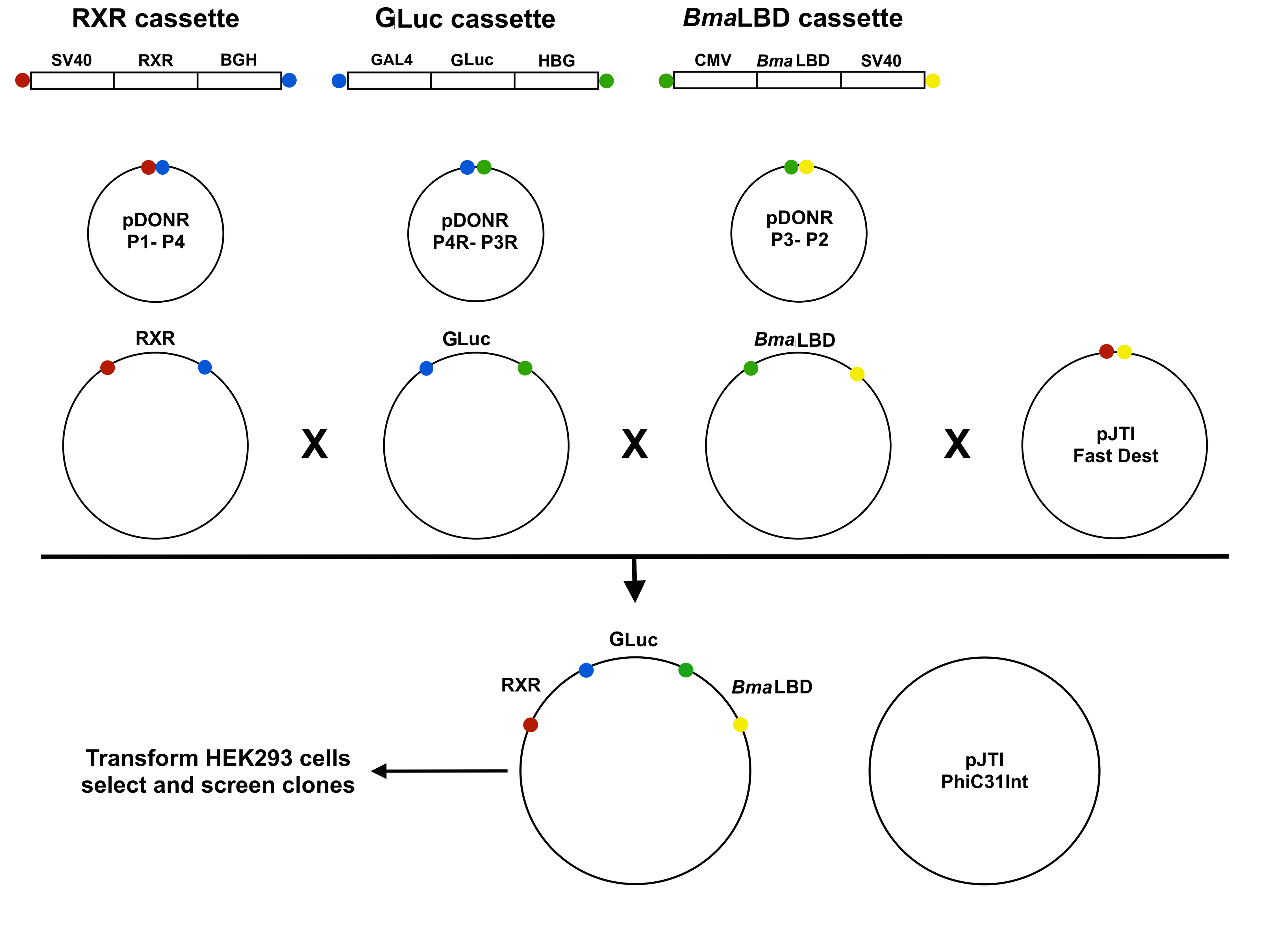

Supplement: S1 Fig — ‘X” = Gateway recombination cloning. Colored circles schematically indicate the specific sequences used in the Gateway recombination cloning reactions. (TIF) [file pntd.0004772.s002.tif]

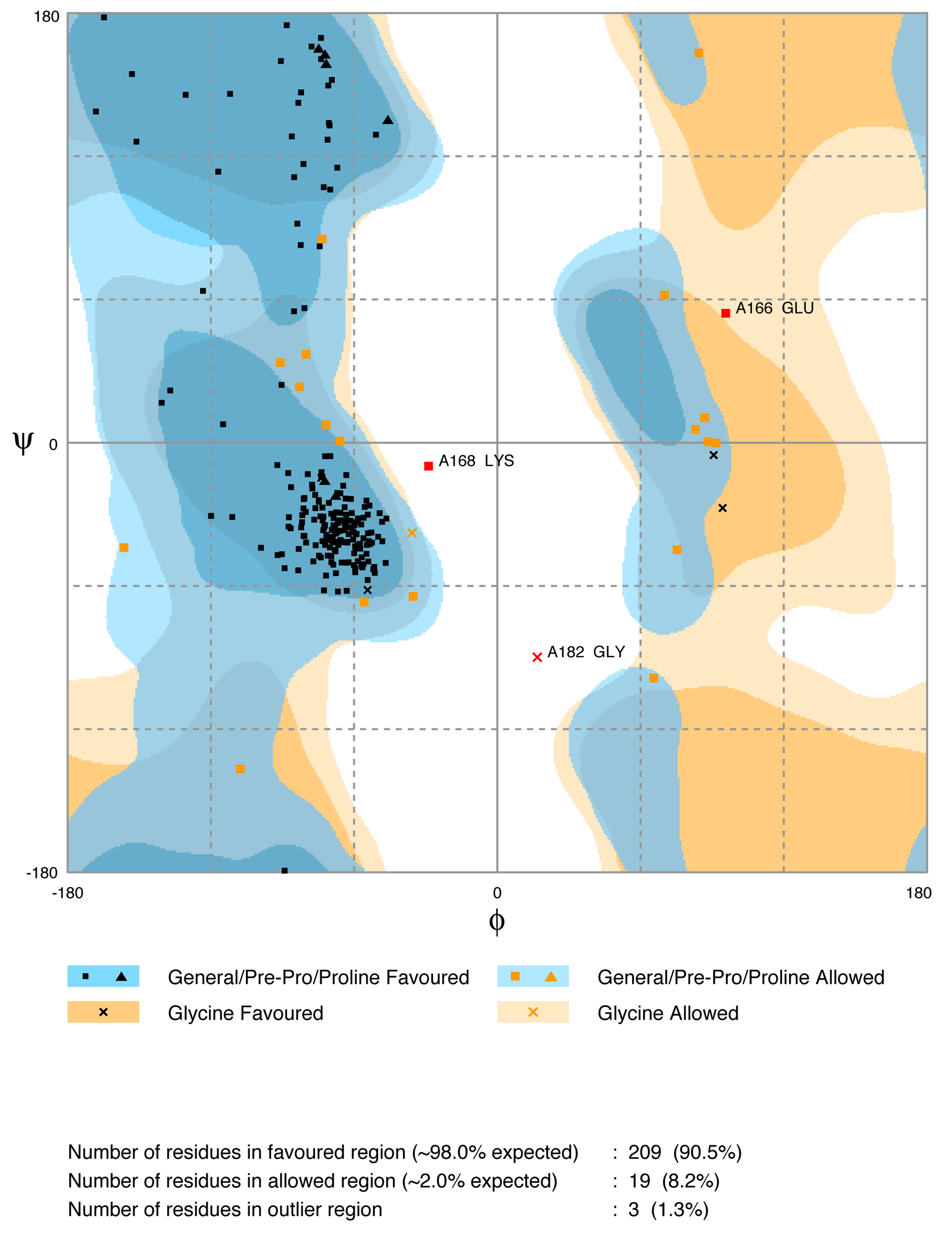

Supplement: S2 Fig — The legend illustrates the color designations of certain regions in the plot. The sum of the first two percentages indicates the number of residues in more favored regions (98.7%). (TIF) [file pntd.0004772.s003.tif]

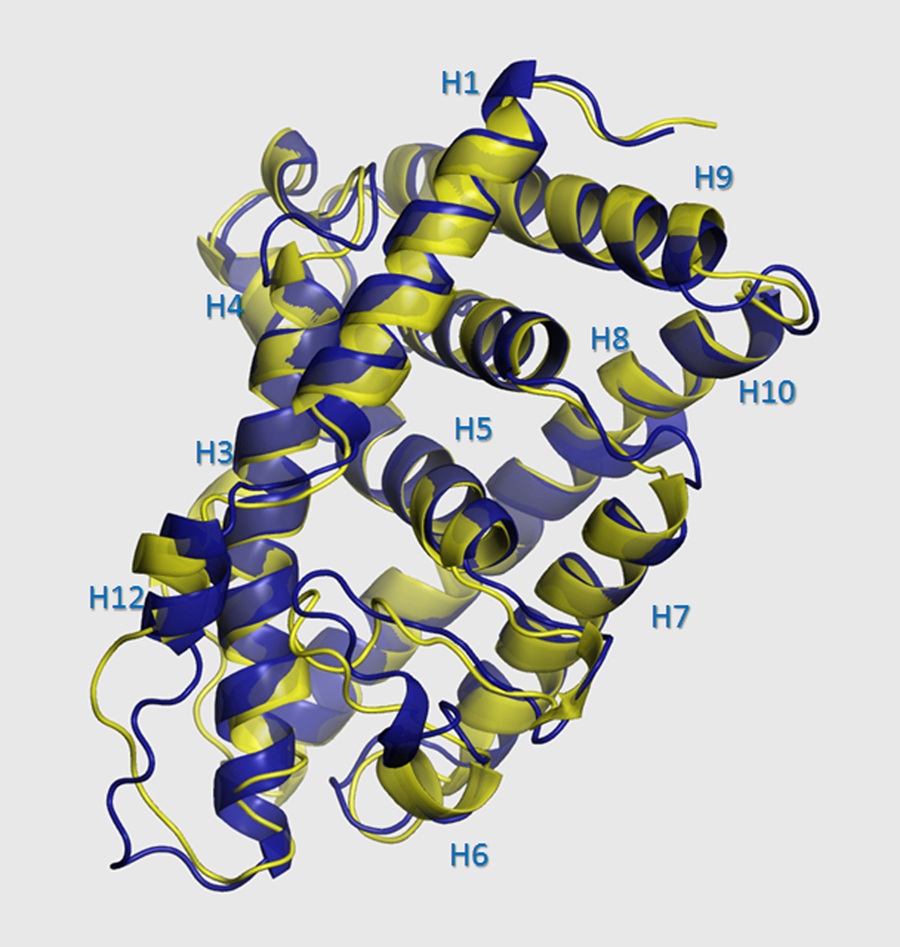

Supplement: S3 Fig — (TIF) [file pntd.0004772.s004.tif]

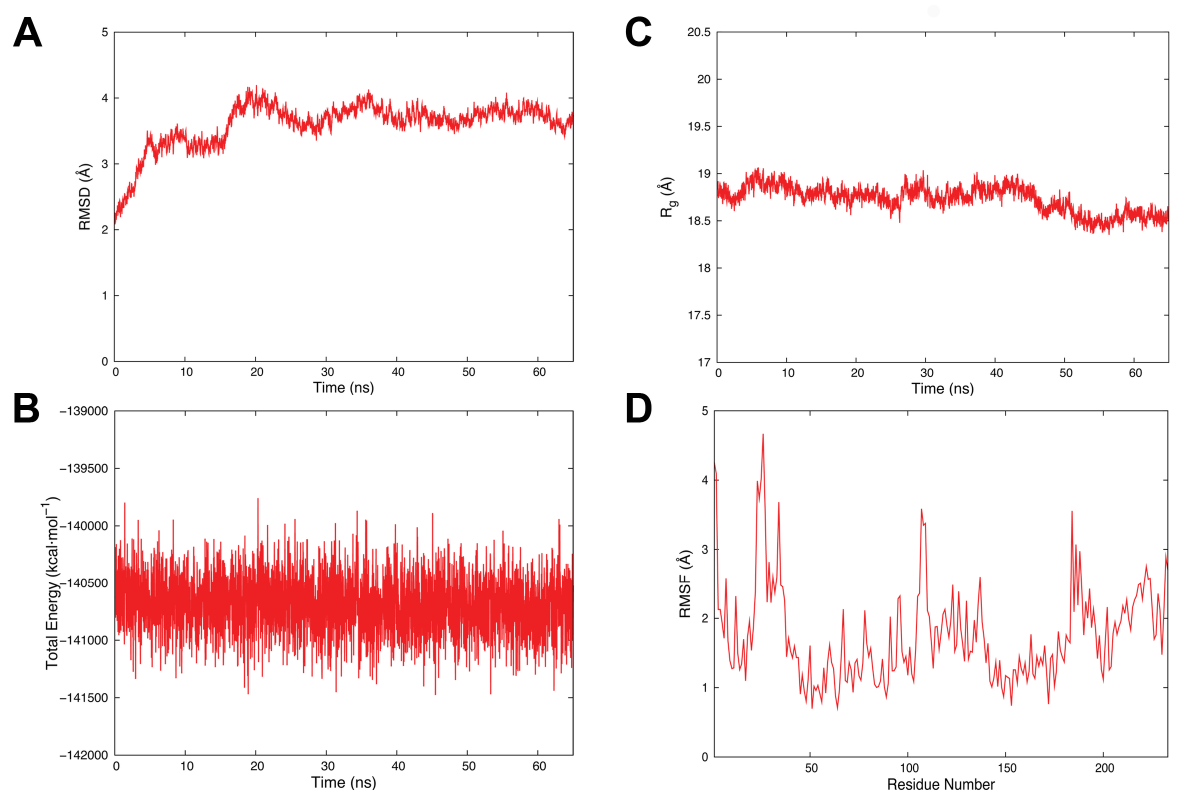

Supplement: S4 Fig — A) RMSD plotted over the course of 65 ns simulation, B) radius of gyration plotted over the course of 65 ns simulation, C) total energy of the homology model and ponasterone A complex monitored over the course of 65 ns simulation, and D) RMSF analysis for each residue in the EcR LBD homology model. (TIF) [file pntd.0004772.s005.tif]

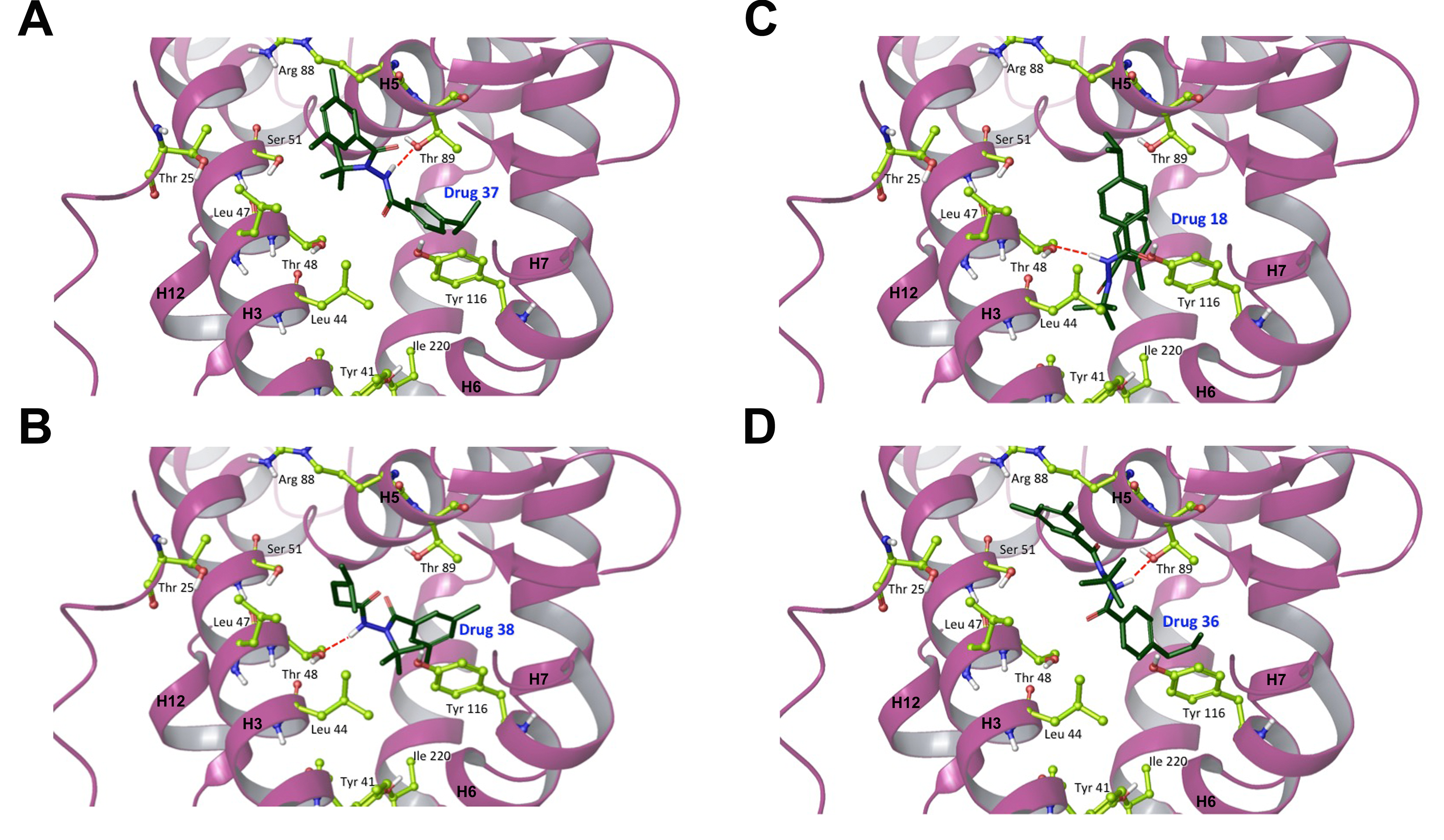

Supplement: S5 Fig — Panel A: Compound 37. Panel B: Compound 38. Panel C: Compound 18. Panel D: Compound 36. (TIF) [file pntd.0004772.s006.tif]
