# Supplementary material for: Identification of Ecdysone Hormone Receptor Agonists as a Therapeutic Approach for Treating Filarial Infections
Source: PLoS Negl Trop Dis. 2016 Jun 14;10(6):e0004772. doi: 10.1371/journal.pntd.0004772 (PMC4907521; doi:10.1371/journal.pntd.0004772)
Supplement: S1 Table — Residue name is given on the left of each column and the RMSF is given to on the right. Residues are ordered by their residue number. Residues constituting the “hydrophobic pocket” are underlined. (DOCX) [file pntd.0004772.s007.docx]

**S1 Table:** **Summary of RMSFs calculated from the MD simulation of for all residues within 3 Å of the 20-hydroxyecdysone binding site.** Residue name is given on the left of each column and the RMSF is given to on the right. Residues are ordered by their residue number. Residues constituting the “hydrophobic pocket” are underlined.

| Residue | RMSF(Å) | Residue | RMSF(Å) | Residue | RMSF(Å) | Residue | RMSF(Å) |
| --- | --- | --- | --- | --- | --- | --- | --- |
| Thr25 | 4.02 | Leu54 | 1.03 | Gly103 | 1.09 | Ala124 | 1.52 |
| Tyr41 | 1.43 | Leu85 | 1.02 | Leu102 | 1.20 | Met127 | 1.86 |
| Leu44 | 1.43 | Arg88 | 1.01 | Tyr116 | 1.56 | Phe128 | 1.64 |
| Leu47 | 0.95 | Thr89 | 0.87 | Phe119 | 2.13 | Ile220 | 2.28 |
| Thr48 | 0.85 | Cys92 | 1.24 | Ile120 | 1.90 | Ile222 | 2.75 |
| Ser51 | 0.70 | Val100 | 1.18 | Ile123 | 2.49 |  |  |
