# Supplementary material for: Identification of Ecdysone Hormone Receptor Agonists as a Therapeutic Approach for Treating Filarial Infections
Source: PLoS Negl Trop Dis. 2016 Jun 14;10(6):e0004772. doi: 10.1371/journal.pntd.0004772 (PMC4907521; doi:10.1371/journal.pntd.0004772)
Supplement: S2 Table — (DOCX) [file pntd.0004772.s008.docx]

**S2 Table:** **Summary of XP energy decomposition analyses**

|  | XP Descriptors | | | | | |
| --- | --- | --- | --- | --- | --- | --- |
| Compound | E_Hbond_ | E_PhobEn_ | Rew_Low MW_ | E_Lipophilic_ | E_Electro_ | E_Penalties_ |
| 20-Hydroxyecdysone | -1.18 | 0 | -- | -5.26 | -0.86 | 0.26 |
| Muristerone A | -2.16 | -0.56 | -- | -4.71 | -0.87 | 0.17 |
| Ponasterone A | -2.55 | -0.68 | -- | -5.43 | -0.75 | 0.23 |
| Diacylhydrazine # 28 | -0.57 | -2 | -0.28 | -5 | -0.29 | 0.18 |
| Diacylhydrazine # 37 | -0.51 | -1.3 | -0.29 | -5.35 | -0.14 | 0.47 |
| Diacylhydrazine # 18 | -0.54 | -0.97 | -0.26 | -5.3 | -0.24 | 0.24 |
| Diacylhydrazine # 38 | -0.65 | -0.14 | -0.44 | -4.87 | -0.19 | 0.47 |
| Diacylhydrazine # 36 | -0.7 | -0.87 | -0.28 | -4.65 | -0.11 | 0.59 |
