# Supplementary material for: Identification of Ecdysone Hormone Receptor Agonists as a Therapeutic Approach for Treating Filarial Infections
Source: PLoS Negl Trop Dis. 2016 Jun 14;10(6):e0004772. doi: 10.1371/journal.pntd.0004772 (PMC4907521; doi:10.1371/journal.pntd.0004772)
Supplement: S3 Table — (DOCX) [file pntd.0004772.s009.docx]

**S3 Table: Compounds identified by the *in-silico* virtual screening and their docking scores.**

| **PubChem/ Chemspider ID** | **IUPAC name** | **Docking Score** |
| --- | --- | --- |
| 5287509 | 2-[3,5-dibromo-4-[4-hydroxy-3-[hydroxy-(2-phenylethylamino)methyl]phenoxy]phenyl]ethane-1,1-diol | -14.7 |
| 49837867 | 4-[[(2S)-2-[2-(4-chlorophenyl)-5,6-difluorobenzimidazol-1-yl]-2-cyclohexylacetyl]amino]-3-methylbenzoic acid | -13.31776 |
| 56603803 | (2R)-2-[4-[3-[4-[(2R)-2-hydroxy-3,3-dimethylbutoxy]-3-methylphenyl]pentan-3-yl]-2-methylphenoxy]butane-1,4-diol | -12.93604 |
| 16214849 | 6-[4-[[3-(2,6-dichlorophenyl)-5-propan-2-yl-1,2-oxazol-4-yl]methoxy]phenyl]naphthalene-1-carboxylic acid | -12.65447 |
| 445460 | 3-fluoro-4-[[(2S)-2-hydroxy-2-(5,5,8,8-tetramethyl-6,7-dihydronaphthalen-2-yl)acetyl]amino]benzoic acid | -12.346 |
| 25166350 | 3-[6-[[3-(2,6-dichlorophenyl)-5-propan-2-yl-1,2-oxazol-4-yl]methoxy]-1-benzothiophen-2-yl]benzoic acid | -12.34432 |
| 9909190/  BMS 493 | 4-[(E)-2-[5,5-dimethyl-8-(2-phenylethynyl)-6H-naphthalen-2-yl]ethenyl]benzoic acid | -12.2792 |
| 10436120 | (1R,3S,5Z)-5-[(2E)-2-[(1R,3aS,7aR)-1-(2,10-dihydroxy-2,10-dimethylundecan-6-yl)-7a-methyl-2,3,3a,5,6,7-hexahydro-1H-inden-4-ylidene]ethylidene]-4-methylidenecyclohexane-1,3-diol | -12.08685 |
| 6398761  Maxacalcitol | (1R,3S,5Z)-5-[(2E)-2-[(1S,3aS,7aS)-1-[(1S)-1-(3-hydroxy-3-methylbutoxy)ethyl]-7a-methyl-2,3,3a,5,6,7-hexahydro-1H-inden-4-ylidene]ethylidene]-4-methylidenecyclohexane-1,3-diol | -11.69114 |
| 146693  22β-Hydroxycholesterol | 3S,8S,9S,10R,13S,14S,17R)-17-[(2S,3R)-3-hydroxy-6-methylheptan-2-yl]-10,13-dimethyl-2,3,4,7,8,9,11,12,14,15,16,17-dodecahydro-1H-cyclopenta[a]phenanthren-3-ol | -11.60698 |
| 11352536 | 2-[4-[butyl-[(3-chloro-4,5-dimethoxyphenyl)methyl]amino]phenyl]-1,1,1,3,3,3-hexafluoropropan-2-ol | -11.49709 |
| 5289548 | 1R,3R)-5-[(2E)-2-[(1R,3aS,7aR)-1-[(2R)-6-hydroxy-6-methylheptan-2-yl]-7a-methyl-2,3,3a,5,6,7-hexahydro-1H-inden-4-ylidene]ethylidene]-2-methylcyclohexane-1,3-diol | -11.38218 |
| 9935197 | 2-[3-chloro-4-[3-[[7-propyl-3-(trifluoromethyl)-1,2-benzoxazol-6-yl]oxy]propylsulfanyl]phenyl]acetic acid | -11.37474 |
| 4469124 | 6-[hydroxy-(5,5,8,8-tetramethyl-6,7-dihydronaphthalen-2-yl)methyl]naphthalene-2-carboxylic acid | -11.35766 |
| 5289501/  TTNPB | 4-[(E)-2-(5,5,8,8-tetramethyl-6,7-dihydronaphthalen-2-yl)prop-1-enyl]benzoic acid | -11.05898 |
| 5288670/  Lexacalcitol | (1R,3S,5Z)-5-[(2E)-2-[(1S,3aS,7aS)-1-[(1R)-1-(4-ethyl-4-hydroxyhexoxy)ethyl]-7a-methyl-2,3,3a,5,6,7-hexahydro-1H-inden-4-ylidene]ethylidene]-4-methylidenecyclohexane-1,3-diol | -11.0335 |
| 44192388 | (1R,3S,5Z)-5-[(2E)-2-[(1R,3S,3aS,7aR)-1-[(2R)-6-hydroxy-6-methylheptan-2-yl]-3-methoxy-7a-methyl-2,3,3a,5,6,7-hexahydro-1H-inden-4-ylidene]ethylidene]-4-methylidenecyclohexane-1,3-diol | -10.9312 |
| 44141919 | 4-[2-(1,1,3,3-tetramethyl-2H-inden-5-yl)-1,3-dioxolan-2-yl]benzoic acid | -10.80572 |
| 46901277 | (1R,3R)-5-[(2E)-2-[(1R,3aS,7aR)-1-[(2R)-5-hydroxypentan-2-yl]-7a-methyl-2,3,3a,5,6,7-hexahydro-1H-inden-4-ylidene]ethylidene]-2-methylidenecyclohexane-1,3-diol | -10.75546 |
| 2126/  AM580 | 4-[(5,5,8,8-tetramethyl-6,7-dihydronaphthalene-2-carbonyl)amino]benzoic acid | -10.68504 |
| 2418 | 3-fluoro-4-[[2-hydroxy-2-(5,5,8,8-tetramethyl-6,7-dihydronaphthalen-2-yl)acetyl]amino]benzoic acid | -10.49947 |
| 56844264 | N-methyl-N-[4-[(1S)-2,2,2-trifluoro-1-hydroxy-1-[1-(2-methoxyethyl)pyrrol-2-yl]ethyl]phenyl]benzenesulfonamide | -10.07298 |
| 49817357 | (2S)-2-[2-(4-chlorophenyl)benzimidazol-1-yl]-N,2-dicyclohexylacetamide | -9.211291 |
| 44141920 | 4-[2-(1,1,3,3-tetramethyl-2H-1,3-benzodisilol-5-yl)-1,3-dioxolan-2-yl]benzoic acid | -8.99854 |
| 10180805 | N-[2-(2-chlorophenyl)-4-methyl-5-propan-2-ylimidazol-1-yl]-5-methyl-2,3-dihydro-1,4-benzodioxine-6-carboxamide | -7.881713 |
