# Supplementary material for: Identification of Ecdysone Hormone Receptor Agonists as a Therapeutic Approach for Treating Filarial Infections
Source: PLoS Negl Trop Dis. 2016 Jun 14;10(6):e0004772. doi: 10.1371/journal.pntd.0004772 (PMC4907521; doi:10.1371/journal.pntd.0004772)
Supplement: S4 Table — (DOCX) [file pntd.0004772.s010.docx]

**S4 Table: Molting of L3 stage larvae when treated with 20-hydroxyecdysone**

|  | **Average days to molt** |
| --- | --- |
| **20E Treated** | 7.584 ± 0.669* |
| **Control** | 11.25 ± 0.966* |

P < 0.001; Student’s t test
