# Supplementary material for: Identification of Ecdysone Hormone Receptor Agonists as a Therapeutic Approach for Treating Filarial Infections
Source: PLoS Negl Trop Dis. 2016 Jun 14;10(6):e0004772. doi: 10.1371/journal.pntd.0004772 (PMC4907521; doi:10.1371/journal.pntd.0004772)
Supplement: S5 Table — (DOCX) [file pntd.0004772.s011.docx]

**S5 Table: Comparison of the transient transfection assay when conducted with NIH3T3 and HEK293 cells.**

|  | **S/N ratio** | **Z'** |
| --- | --- | --- |
| NIH3T3 | 6.29 | 0.816 |
| HEK293 | 8.31 | 0.857 |
